# Decoding the future from past experience: learning shapes predictions in early visual cortex

**DOI:** 10.1152/jn.00753.2014

**Published:** 2015-03-05

**Authors:** Caroline D. B. Luft, Alan Meeson, Andrew E. Welchman, Zoe Kourtzi

**Affiliations:** ^1^Department of Psychology, Goldsmiths, University of London, London, United Kingdom;; ^2^School of Psychology, University of Birmingham, Birmingham, United Kingdom; and; ^3^Department of Psychology, University of Cambridge, Cambridge, United Kingdom

**Keywords:** visual learning, prediction, fMRI, visual cortex, sensory processing

## Abstract

Learning the structure of the environment is critical for interpreting the current scene and predicting upcoming events. However, the brain mechanisms that support our ability to translate knowledge about scene statistics to sensory predictions remain largely unknown. Here we provide evidence that learning of temporal regularities shapes representations in early visual cortex that relate to our ability to predict sensory events. We tested the participants' ability to predict the orientation of a test stimulus after exposure to sequences of leftward- or rightward-oriented gratings. Using fMRI decoding, we identified brain patterns related to the observers' visual predictions rather than stimulus-driven activity. Decoding of predicted orientations following structured sequences was enhanced after training, while decoding of cued orientations following exposure to random sequences did not change. These predictive representations appear to be driven by the same large-scale neural populations that encode actual stimulus orientation and to be specific to the learned sequence structure. Thus our findings provide evidence that learning temporal structures supports our ability to predict future events by reactivating selective sensory representations as early as in primary visual cortex.

successful everyday interactions entail that we exploit information about the structure of the environment to interpret the current scene and predict upcoming events. Recent theoretical work (Geisler 2008; Petrov et al. 2005) suggests that the brain achieves this challenge by learning through exposure to the environment's statistics. There is accumulating evidence that mere exposure to stimuli that co-occur in the environment facilitates our ability to extract spatial and temporal regularities (for reviews, see Aslin and Newport 2012; Perruchet and Pacton 2006). However, the brain mechanisms that mediate our ability to predict upcoming events based on previous knowledge about the environment's statistics remain largely unknown.

Previous neuroimaging work has implicated subcortical and medial temporal lobe regions in the learning of temporal statistics. In particular, the striatum and hippocampus have been implicated in learning of probabilistic associations (Poldrack et al. 2001; Shohamy and Wagner 2008) and temporal sequences (Gheysen et al. 2011; Hsieh et al. 2014; Rauch et al. 1997; Rose et al. 2011; Schapiro et al. 2012, 2014; Schendan et al. 2003a). While these brain regions are thought to be involved at the initial stages of training, prefrontal regions have been shown to engage at later learning stages (Leaver et al. 2009; Pasupathy and Miller 2005). Despite accumulating evidence for neural circuits involved in learning temporal regularities, it remains unknown whether this knowledge of temporal statistics facilitates sensory predictions. Here we tested whether learning of temporal regularities shapes processing in primary visual cortex and mediates our ability to predict the identity of upcoming visual stimuli.

We devised a novel paradigm to measure behavioral performance and brain activity related to visual predictions. First, we tested the participants' ability to predict the identity of a visual stimulus (i.e., grating orientation) after learning of temporal sequences. Our behavioral results demonstrate that observers learn to exploit temporal regularities and improve their ability to predict the identity of upcoming stimuli. Second, using fMRI we tested whether processing in visual cortex is altered after learning of temporal sequences and reflects the observers' improved ability to predict the identity of upcoming stimuli. To ensure that we measured activity related to the observers' predictions rather than the presented stimuli, we introduced a long blank interval between the presentation of temporal sequences and the test stimulus (Fig. 1*A*). Despite the low BOLD signal during this period of no stimulation, we were able to decode the orientation predicted by the observers in each trial after training with multivoxel pattern (MVPA) classification methods. Furthermore, to test whether decoding accuracy reflected knowledge of temporal structure, we tested brain activity before and after training when observers were presented with a random sequence and asked whether a cued orientation at the end of the sequence matched the test stimulus. Decoding of cued orientations following a random sequence did not change after training and was weaker than decoding of predicted orientations following structured sequences, suggesting that learning of temporal structure shapes predictive representations in primary visual cortex.

**Fig. 1.**
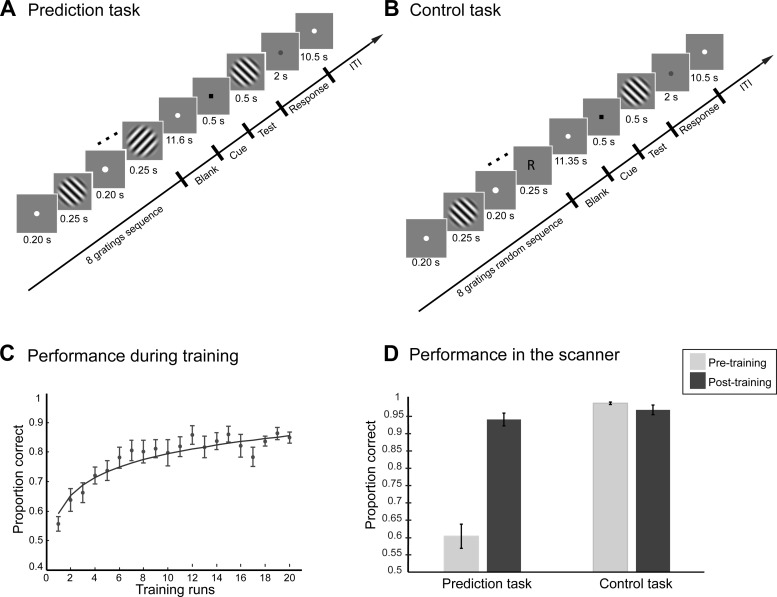
Task design and behavioral performance. *A*: prediction task: in each trial, participants were presented with a structured sequence of 8 gratings followed by a blank interval (11.6 s). After this period, a test grating, preceded by a brief cue (black square, 0.5 s), was presented and the participants had to indicate whether the orientation of the test grating matched their expectation or not. *B*: control task: the participants were instructed to attend to the sequence and indicate whether a cued grating (“R” or “L”) matched the orientation of the test stimulus or not. The timing for this task was matched to the prediction task. *C*: mean proportion of correct responses for each run across training sessions. Data are shown for 12/16 participants who showed improvement after training, excluding participants (*n* = 4) who did not show improvement in the task after training (57% mean performance at the last training session). Data across runs were fitted with least-squares nonlinear fit. Data are shown for 16 blocks (4 training sessions). One participant completed only 3 sessions, as performance had already saturated above 80%; the rest of the participants completed either 4 (*n* = 4) or 5 (*n* = 7) training sessions. *D*: mean proportion of correct responses during scanning before and after training for both prediction and control tasks. Error bars indicate SE. Behavioral data for all 16 participants showed a similar pattern of results; that is, we observed a significant effect of session (*F*_1,11_ = 144.13, *P* < 0.001) and task and session interaction (*F*_1,11_ = 76.08, *P* < 0.001).

## MATERIALS AND METHODS

### Participants

Sixteen undergraduates from the University of Birmingham (mean age 21 ± 2.6 yr) took part in the study. All participants were naive to the aims of the study, were right-handed, had normal or corrected-to-normal vision, had no history of neurological disorders, and gave written informed consent. This study was approved by the University of Birmingham Ethics Committee.

### Stimuli

Stimuli comprised grayscale sinusoidal gratings that were presented at 9° visual angle, spatial frequency that ranged from 0.85 to 1 cycles/° across trials, 100% contrast, and randomized phase. These gratings were rotated ±45° from vertical orientation (90°), resulting in gratings oriented at either 135° (left) or 45° (right). To avoid local position adaptation, we randomized the phase and jittered the orientation within a range of 2° across trials. We used these stimuli to generate two sequences, each comprising eight gratings, as shown below (1 refers to a leftward-oriented grating and 2 refers to a rightward-oriented grating):

sequence A: 2 1 2 1 1 2 1 2

sequence B: 1 1 2 1 2 2 1 2

As the two sequences predicted different orientations (*sequence A* predicted a rightward-oriented grating; *sequence B* predicted a leftward-oriented grating), we generated two more sequences by replacing leftward with rightward orientations and vice versa while keeping the sequence structure the same. These sequences were as follows:

sequence A′=1 2 1 2 2 1 2 1

sequence B′=2 2 1 2 1 1 2 1

This manipulation allowed us to counterbalance for the predicted orientations, as it resulted in sequences that had the same structure but predicted different orientations (e.g., *A* and *A′*) and sequences that had different structures but predicted the same orientation (e.g., *A* and *B′*). This ensured that decoding of the predicted orientation was not confounded by the specific orientations used at each temporal position but related to knowledge of the sequence structure. Analysis of the behavioral data showed that the participants were equally accurate across all sequences. This was confirmed by a 2 (sequence type: *A* vs. *B*) × 2 (sequence version: *A*/*B* vs. *A′*/*B′*) repeated-measures ANOVA, which showed that there was no significant effect for sequence type (*F*_1,11_ = 0.08, *P* = 0.783) or sequence version (*F*_1,11_ = 2.17, *P* = 0.169) nor a significant interaction (*F*_1,11_ = 0.06, *P* = 0.816).

Each sequence comprised four leftward- and four rightward-oriented gratings. As all gratings were presented at the same rate, participants could not use stimulus duration to group elements together or segment the sequences. Furthermore, to ensure that participants did not perform the task simply by memorizing the first stimulus in the sequence, the orientation of the first stimulus was randomized in each trial during scanning; that is, for each of the four sequences half of the trials started with leftward-oriented and the rest with rightward-oriented gratings. Also, to ensure that participants did not learn the task simply by memorizing the last orientations in the sequence, the last three stimuli in each sequence pair (*A* and *B*; *A′* and *B′*) were the same across all sequences. These manipulations preserved equal frequency of appearance for the two orientations across trials. Finally, as the frequency of occurrence was matched for the two grating orientations in the sequence and the participants did not know how many items each sequence contained, to perform the task participants were required to learn the order of the elements in the sequence (i.e., temporal order associations among pairs or triplets of oriented gratings).

Stimuli were generated and presented with Psychtoolbox-3 (Brainard 1997). For the behavioral training sessions, stimuli were presented on a 21-in. CRT monitor (ViewSonic P225f 1,280 × 1,024 pixels, 85 Hz frame rate) at a distance of 45 cm. For the pre- and posttraining fMRI scans, stimuli were presented with a projector and a mirror setup (1,280 × 1,024 pixels, 60 Hz frame rate) at a viewing distance of 64 cm. To keep the same visual angle for both training and scanning sessions, the stimulus size was adjusted according to the viewing distance.

### Design

All participants (*n* = 16) took part in two pretraining fMRI sessions, three to five behavioral training sessions, and two posttraining fMRI sessions. Participants were tested in both the prediction task (1 pretraining and 1 posttraining scan) and the control task (1 pretraining and 1 posttraining scan). The order of the scans (prediction vs. control task) was counterbalanced across participants. After the pretraining scans, all participants were trained for three to five sessions in the lab. The number of training sessions was determined by each participant's performance: the training stopped when the participant reached performance higher than 80% correct in all four runs comprising a training session. The posttraining scans took place on subsequent days after the last behavioral training session (one scan in the first and the other in the second day after the last training session). In addition, the participants completed two scanning runs of an orientation decoding experiment and two retinotopy localizer scans.

### Behavioral Training

Participants were trained on the prediction task without feedback for three to five sessions. Participants viewed 16 gratings (each sequence of 8 gratings was repeated twice in a trial) presented sequentially on a gray background at the center of the screen. All stimuli were presented at the same rate; that is, each grating was presented for 0.3 s, followed by a fixation interval of 0.3 s. Participants were asked to respond to a test grating that appeared for 0.3 s surrounded by a red circle (0.3 s). The test stimulus was preceded by a cue (red dot presented for 1 s) and was followed by a white fixation dot (1,700 ms). Participants were instructed to respond (the maximum response time was 2,000 ms), indicating whether the test image had the same orientation (left vs. right) as the grating they expected to appear in that position in the sequence. The test stimulus appeared only in the second repeat of the sequence, and its position was randomized across trials. The test stimulus could appear in any position in the sequence except the last three positions; stimuli in these positions were the same across trials. For each run, 50% of the test stimuli were presented at the correct orientation for their position in the sequence. After the participant's response, the remaining gratings in the sequence were presented until all 16 stimuli had been presented, ensuring that all trials had the same length. A black cross (1 s) indicated the end of the sequence and the start of a new trial. There was no feedback across all training sessions. In each training session, participants performed the prediction task for 4 runs of 40 trials each (20 per sequence type) with a minimum 2-min break between runs. The number of training sessions was determined on the basis of performance; the participants stopped training after reaching consistent session accuracy above 80% (all training runs within 1 session had to be above 80%).

After each training session, the participants were asked to complete a debriefing questionnaire with the following questions: *1*) Please describe any strategies you may have used when responding to this task; *2*) How many of your responses do you think were correct? (1 to 5, from “few correct” to “most correct”); *3*) How did you find the task? (1 to 5, from “very difficult” to “very easy”); *4*) How tired did you feel at the end of each run? (1 to 5, from “very tired” to “not tired at all”); *5*) How many different sequences of stimuli do you think were presented? In addition, after the last training session, the participants were asked to write down the sequences that they thought were presented during the experiment.

### fMRI Design

The participants took part in two pre- and two post-training scans: one pre- and one posttraining scan for the prediction task and one pre- and one posttraining scan for the control task. The order of the scans was counterbalanced: half of the participants did the prediction task in the first pre- and posttraining scan session, whereas the other half started with the control task. In addition, the participants completed two runs of an orientation decoding experiment and two retinotopy localizer scans (polar angle and eccentricity).

#### Prediction task scan.

Participants completed seven to nine runs (12 trials per run) of the prediction task per scan session (Fig. 1*A*). Each run followed an event-related design comprising 12 trials and a fixation block (15 s) at the beginning and end of the run. Participants were presented with all four sequences used for training. Each sequence was repeated once per trial (comprising 8 stimuli), followed by a test stimulus. For each trial (28.5 s long), a sequence of eight leftward (135°)- or rightward (45°)-oriented gratings was presented. Each grating was presented for 0.25 s, followed by fixation for 0.2 s. The sequence of gratings was followed by a fixation period (11.6 s), a cue (black square, 0.5 s), a test grating (0.5 s), and a red dot (2 s) before the start of the next trial. The participants were instructed to pay attention to the sequence and to respond whether the orientation of the test grating matched the orientation they expected to follow from the preceding sequence. To ensure that all participants viewed the test grating for the same duration and there were no differences in reaction time across participants, participants were instructed to delay their response until the red dot appeared after the test grating. After each trial, there was a fixation period of 10.5 s.

To acquire adequate data (i.e., number of trials) for the fMRI analysis within the time constraints of the scanning sessions, we used shorter sequences (single sequence comprising 8 stimuli) during scanning (instead of 2 repeats of the same 8-item sequence comprising in total 16 stimuli during training). That is, during scanning participants were required to predict the orientation of the stimulus in the ninth temporal position of the trained sequences. Our experimental design during training (i.e., same presentation duration across stimuli, variable temporal test position during training) made it unlikely that the participants had explicit knowledge of the sequence length or number of sequence repeats, as also indicated by debriefing. Furthermore, to ensure that participants did not simply memorize the first stimulus in the sequence during scanning, we randomized (across trials) the orientation of the first stimulus that was then followed by the remaining seven items in the sequence. Thus it is unlikely that participants memorized the orientation of stimuli presented at individual temporal positions for each of the four trained sequences. In contrast, it is more likely that participants learned temporal associations between sequence items (i.e., pairs or triplets) during training that remained the same in the test sequences and facilitated their predictions.

#### Control task scan.

Participants completed seven to nine runs (12 trials per run) of a control task per scan session (Fig. 1*B*). To test learning improvement specific to structured sequences, we presented participants with a random sequence of leftward- and rightward-oriented gratings that were presented equally often but at randomized positions within the sequence. To ensure that performance in the control task was comparable to the prediction task after training, we asked participants to compare the orientation of the test grating with a cued orientation presented after the sequence. This allowed us to compare fMRI activations between tasks (prediction vs. control) with comparable levels of behavioral performance. Each run followed an event-related design comprising 12 trials and a fixation block (15 s) at the beginning and end of the run. The per-trial design for the control task matched that of the prediction task. Each trial (28.5 s long) comprised a random sequence of eight leftward (135°)- or rightward (45°)-oriented gratings (i.e., gratings were presented at random order in the sequence). Each grating was presented for 0.25 s, followed by fixation for 0.2 s. This random sequence of gratings was followed by a cue, “R” or “L” (0.25 s), indicating whether the participants should remember a rightward- or leftward-oriented grating, respectively. This cue was followed by a fixation period (11.35 s), a cue (black square, 0.5 s), a test grating (0.5 s), and a red dot (2 s) before the start of the next trial. The control and prediction tasks were matched on a trial-by-trial basis for the orientation of expected and remembered items; that is, the orientation indicated by the cue in the control task matched the expected orientation in the prediction task on a per-trial basis. The participants were instructed to pay attention to the sequence, remember a grating rotated leftward or rightward as indicated by the cue, and indicate whether the test grating matched the cued orientation.

#### Orientation decoding scan.

All participants completed two runs of an orientation decoding experiment following procedures described previously (Harrison and Tong 2009). Participants were presented with the same gratings as in the prediction and control tasks (100% contrast and oriented either leftward or rightward). Leftward- vs. rightward-oriented gratings were presented in separate 15-s-long blocks. Similar to the prediction task scans, to avoid adaptation due to stimulus repetition, we randomized the phase and jittered the orientation of the gratings within a range of 2° across trials. Each block comprised 30 gratings. Each grating was presented for 0.25 s, followed by a blank interval of 0.25 s. Each run comprised 20 blocks (10 per orientation) and 2 fixation blocks: 1 in the beginning and 1 at the end of the run. The order of the blocks was randomized across runs. Participants were asked to perform a contrast change detection task on the fixation. That is, participants were instructed to press a button when they detected a contrast change at fixation (twice per block at random time points).

#### Retinotopic mapping scans.

For each participant we independently localized regions in the early (V1, V2) and higher (V3v, V3d, and hV4) visual areas, following standard retinotopic mapping procedures (e.g., Sereno et al. 1995). Data from polar and eccentricity scans were collected during either the pre- or posttraining scan session. hV4 comprises the ventral but not the dorsal subregion of V4.

### fMRI Data Acquisition

fMRI data were acquired in a 3-T Achieva Philips scanner at the Birmingham University Imaging Centre using an eight-channel head coil. Anatomical images were obtained with a sagittal three-dimensional T1-weighted sequence (voxel size = 1 × 1 × 1 mm, slices = 175). Functional EPI images were acquired with a high-resolution gradient echo-pulse sequence covering the occipital and posterior temporal cortex [20 slices at 1.5 × 1.5 × 2-mm resolution; matrix size 128 × 128; slice thickness: 2 mm with no gap between slices; FOV: 192 × 192; repetition time (TR) 1,500 ms; time to echo (TE) 35 ms].

### Eye Movement Recordings

We recorded eye movements (*n* = 6) with the ASL 6000 Eye-tracker (Applied Science Laboratories, Bedford, MA; sampling rate: 60 Hz) in the scanner. Eye tracking data were preprocessed with EyeNal Data Analysis software (Applied Science Laboratories) and analyzed with custom toolbox based on MATLAB (MathWorks) software. Because of poor signal quality, data from two participants were excluded from the analysis. Runs with >10% signal loss were removed from the analysis. We computed *1*) horizontal eye position, *2*) vertical eye position, *3*) proportion of saccades for each condition at different saccade amplitude ranges, and *4*) number of saccades per trial per condition during the blank interval following the sequence presentation, separately for each pretraining (*I*) and posttraining (*II*) session. Histograms of the horizontal and vertical eye positions peaked and were centered on the fixation at 0°, suggesting that participants could fixate well both before and after training when predicting leftward- or rightward-oriented gratings.

### Data Analysis

#### Behavioral data analysis.

Performance on the task was assessed by the accuracy in correctly predicting whether the next grating in the sequence was left or right. For the training sessions, we averaged the accuracy for each run of the sequential sessions and estimated a learning rate by fitting a logarithmic function to the data (Fig. 1*C*). Data across runs were fitted (least-squares nonlinear fit) with the following equation: *y* = *k* × log(*x*) + *c*, where *k* is the value of the curve tangent at *x* = 1 and *c* is the value of *y* for *x* = 1.

#### fMRI data preprocessing.

Neuroimaging data were analyzed with Brain Voyager QX (Brain Innovation, Maastricht, The Netherlands). Anatomical data were used for three-dimensional cortex reconstruction, inflation, and flattening. Preprocessing of functional data included slice scan time correction, three-dimensional motion correction, linear trend removal, and temporal high-pass filtering (3 cycles). fMRI data were recorded at high resolution (1.5. × 1.5 mm in plane) and interpolated to 2 × 2 × 2 mm with trilinear interpolation. Trials with head motion larger than 1 mm of translation or 1° of rotation or sharp motion above 0.5 mm (on average 25 trials per session across areas and tasks) were excluded from the analysis. Runs whose motion analysis resulted in the exclusion of >50% of the trials were excluded from further analysis. The functional images were manually aligned to anatomical data, and the complete data were transformed into Talairach space. For each observer, the functional imaging data between the four sessions were coaligned, registering all the volumes for each observer to the first functional volume of the first run and session. The retinotopic mapping scans (polar and eccentricity) were also coaligned with the first volume of the first run and session. This procedure ensured a cautious coregistration across sessions.

#### fMRI decoding.

We used a linear support vector machine (SVM) with a leave-one-run-out cross-validation procedure for pattern classification. To investigate the link between fMRI activity and the participants' responses, we tested the classifier's performance in decoding the participant's prediction (leftward vs. rightward); that is, if the participant responded that a leftward-oriented test grating was “correct,” they predicted “left,” if the participant indicated “incorrect,” then they predicted “right,” and vice versa. That is, we trained the classifier to associate fMRI signals with a label (predicted left vs. predicted right) as indicated by the participant's response to the test grating in each trial. To control for potential bias in the classification due to the unequal numbers of trials responded to as “correct” or “incorrect” by the participants, we used a cost factor and weighted the error term during SVM training by the ratio of fMRI patterns related to “correct response” over fMRI patterns related to “incorrect response.”

To select voxels for the pattern classification analysis, we used the retinotopic mapping scans for each participant. We selected voxels that corresponded to a stimulus area of 8° of visual angle and were significantly more activated by the grating stimuli than the fixation (*P* < 0.05, uncorrected). This procedure allowed us to avoid voxels corresponding to the edges of the grating stimulus (Harrison and Tong 2009). Each voxel time course was *z*-score normalized for each experimental run separately. The data pattern for each trial was generated by shifting the fMRI time series by 3 volumes (4.5 s) to account for the hemodynamic delay. That is, volumes corresponding to the no-stimulation period following the sequence, *volumes 4–10*, were shifted to *volumes 7–13* (Fig. 3 shows fMRI volumes before shifting to account for the hemodynamic delay). We trained and tested the classifier during this no-stimulation period (*volumes 8–13*), excluding the first volume (*volume 7*) to avoid interference from BOLD responses related to the preceding sequence. We performed this analysis on each of these volumes separately as well as on the average signal across these volumes. For each cross-validation (from 5 to 9 depending on the number of runs per participant), we used 60–108 patterns (for averaged signals across no-stimulation volumes) for training the classifier and 12 independent patterns for testing the classifier's accuracy. We plotted classifier accuracy across voxels—starting with voxels that have the highest *t*-value for gratings vs. fixation—and selected the 300 most activated voxels in each region of interest (ROI) for each participant, as pattern classification accuracy had saturated at this pattern size across areas. We then averaged the classifier's accuracy for this pattern size across cross-validations for each participant.

#### Generalization of classifier accuracy.

To evaluate the correspondence between neural representations for physical and predicted orientations we followed two different approaches. First, we identified common voxels across experiments (i.e., informative voxels for both the classification of the physical and the predicted orientation). That is, using a recursive feature elimination (RFE) procedure (Ban et al. 2012; De Martino et al. 2008) we identified voxels across visual areas that contributed (i.e., as indicated by the classifier's linear weights) to the decoding of *1*) the physical stimulus and *2*) the predicted stimulus orientations using data from the orientation decoding experiment and the prediction task, respectively. These two RFE analyses were conducted separately (i.e., the classifiers were trained with a cross-validation procedure on either the physical or the predicted orientations), resulting in two sets of voxels: voxels informative for the classification of the physical stimulus and voxels informative for the classification of the predicted stimulus. We then ranked voxels in each visual area resulting from the two RFE analyses and chose voxels that were informative in both analyses (i.e., 300 most informative voxels). We used these voxels to train an SVM classifier on fMRI signals related to physical orientations and tested the accuracy of this classifier in decoding predicted orientations from fMRI data collected when observers performed the prediction task (i.e., no-stimulation interval following the sequence presentation). It is important to note that we ran the RFE analysis and selected voxels separately for each cross-validation of the MVPA analysis (i.e., decoding of predicted orientations) to avoid circularity; that is, we excluded the test data from both the RFE and the decoding analysis. The results from this analysis are presented in Fig. 5. Second, using the same RFE procedure we identified the top 300 voxels that contributed (i.e., as indicated by the classifier's linear weights) to the decoding of the physical stimulus using data from the orientation decoding experiment only. We then used these voxels to train and test the classifier (using a leave-one-run-out cross-validation procedure) in decoding predicted orientations from fMRI data collected when observers performed the prediction task (i.e., no-stimulation interval following the sequence presentation). The results from the second procedure were similar to those reported in Fig. 3, suggesting that the generalization of classification accuracy that we observed could not be simply due to the voxel selection or MVPA procedure used.

## RESULTS

### Behavioral Results

We presented participants with a sequence of leftward- and rightward-oriented gratings (Fig. 1*A*) and asked them to predict the next grating in the sequence. We trained participants on this prediction task without feedback for three to five sessions (as determined by individual performance). To control for the possibility that observers memorized specific items in the sequence or full sequences rather than learning the temporal structure, we trained participants with four different sequences and presented all stimuli at the same rate and in a continuous stream. Furthermore, the position of the test stimulus was randomized across trials, the last three items were the same across sequences, and for half of the trials the incorrect test stimulus was presented.

Before and after training, we tested participants while they performed the prediction task and a control task. In particular, for the prediction task we asked participants to indicate whether the orientation of a test grating matched the orientation they anticipated following a preceding structured sequence of gratings (Fig. 1*A*). For the control task participants were asked to indicate whether the orientation of a test stimulus matched the orientation of a cued grating presented after a random sequence of oriented gratings (Fig. 1*B*).

Performance on the prediction task improved for most participants (12/16 participants improved; 4 participants did not improve during training, showing 57% mean performance at the last training session) as they gained more exposure to the temporal sequences (Fig. 1*C*). We further focus on the analysis of the behavioral and fMRI data for the 12 participants that showed improvement during training and posttraining performance higher than 80% correct; the data of the weaker learners (posttraining performance lower than 65% correct) are considered below in a separate control analysis. Comparison of performance in the prediction and control tasks during scanning (Fig. 1*D*) showed that observers' performance improved after training in the prediction task, while performance in the control task remained high both before and after training. These results were confirmed by a 2 (task: prediction vs. control task) × 2 (session: pre- vs. posttest) repeated-measures ANOVA showing a significant interaction between task and session (*F*_1,11_ = 76.08, *P* < 0.001), consistent with enhanced performance after training in the prediction (*t*_11_ = −8.50, *P* < 0.001) but not the control (*t*_11_ = 1.42, *P* = 0.183) task. The comparable behavioral performance after training for the prediction and control tasks, which involved structured vs. random sequences respectively, ensured that comparing fMRI activation patterns between the two tasks reflected learning specific to the sequence structure that was not confounded by differences in behavioral performance.

Improvement in the prediction task after training indicates that participants acquired knowledge of the sequence structure. Debriefing the participants suggests that this knowledge was most likely implicit and it was unlikely that the participants memorized the sequences explicitly. In particular, participants were significantly more confident in their responses after training (*t*_11_ = −5.03, *P* < 0.001) and found the task easier (*t*_11_ = −4.31, *P* = 0.001) but did not feel significantly more or less tired (*t*_11_ = −1.48, *P* = 0.166). Interestingly, this was not the case for weaker learners, who did not report any substantial changes in their confidence (pretraining mean = 1.5, SD = 0.58; posttraining mean = 1.75, SD = 0.96) or task difficulty (pretraining mean = 3.50, SD = 1.29; posttraining mean = 2.25, SD = 0.5). Finally, we asked participants to estimate the number of sequences presented during the experiment; this number did not change significantly after training (*t*_11_ = −0.41, *P* = 0.689). Only three participants indicated that there were four sequences in total, but none of the participants could explicitly report the sequences correctly. Taken together, the debriefing data suggest that it was unlikely that the participants improved in the task by explicitly memorizing the sequences.

### fMRI Results: Decoding Predictions in Visual Cortex

**Fig. 2.**
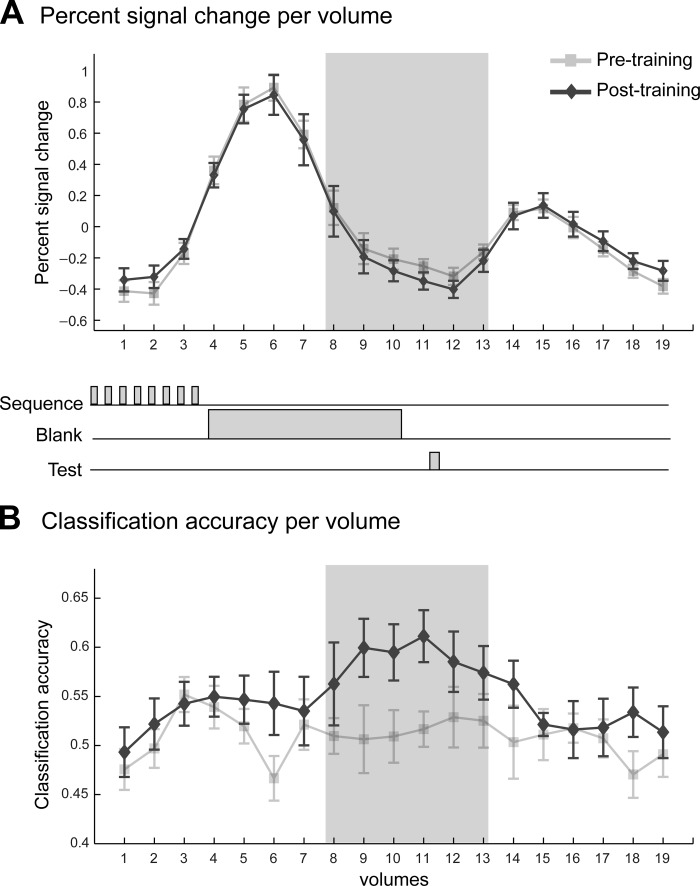
Univariate vs. multivariate analysis of BOLD signals. *A*: univariate mean trial time course (across voxels and participants) of BOLD responses in V1 (% signal change calculated in relation to the average signal across the whole run) for the pretraining and posttraining scanning sessions. The shaded gray area (*volumes 8–13*) indicates the volumes used to decode the participants' prediction after accounting for the hemodynamic lag. *Volume 7* was not used, to avoid confounding activation from the sequence presentation. *B*: mean decoding support vector machine (SVM) accuracy (proportion correct) per fMRI volume of the participants' predictions before and after training. Note that per-volume fMRI signals are noisier than signals averaged across volumes, resulting in lower multivoxel pattern (MVPA) accuracy. Error bars indicate SE.

To test whether learning of temporal regularities shapes sensory predictions in visual cortex, we scanned participants before and after training. For each participant we identified retinotopic visual areas with high-resolution fMRI and standard mapping procedures. To test for fMRI signals related to the participants' predictions rather than the stimuli per se, we extracted activity during a blank interval between the temporal sequences and the test stimulus. Analysis of univariate fMRI signals (percent signal change from mean BOLD response across trials) confirmed that BOLD responses during this no-stimulation period were low and did not differ before vs. after training (paired *t*-test for all ROIs, *P* > 0.05; Fig. 2*A*). However, previous studies have shown that neural preference for orientation can be decoded from no-stimulation intervals in the visual cortex with MVPA classification analysis (Harrison and Tong 2009; Kamitani and Tong 2005; Serences et al. 2009; Tong and Pratte 2012). With this approach, we trained an SVM classifier to associate responses from each fMRI volume to the participants' prediction in each trial and tested the accuracy of the classifier in predicting the participants' responses (leftward vs. rightward predicted orientation) using an independent data set. In contrast to the univariate signals, this analysis allowed us to successfully decode the orientation predicted by the participants from fMRI signals during periods of no stimulation in V1. Importantly, classification accuracy increased after compared with before training (Fig. 2*B*). Inspection of the MVPA accuracy time course across volumes showed that the improvement in decoding performance after training peaked during the blank interval following the presentation of the temporal sequence during which participants predicted the orientation of the upcoming stimulus (Fig. 2*B*). This result reflects learning-dependent changes specific to processing of predicted orientations in the primary visual cortex.

**Fig. 3.**
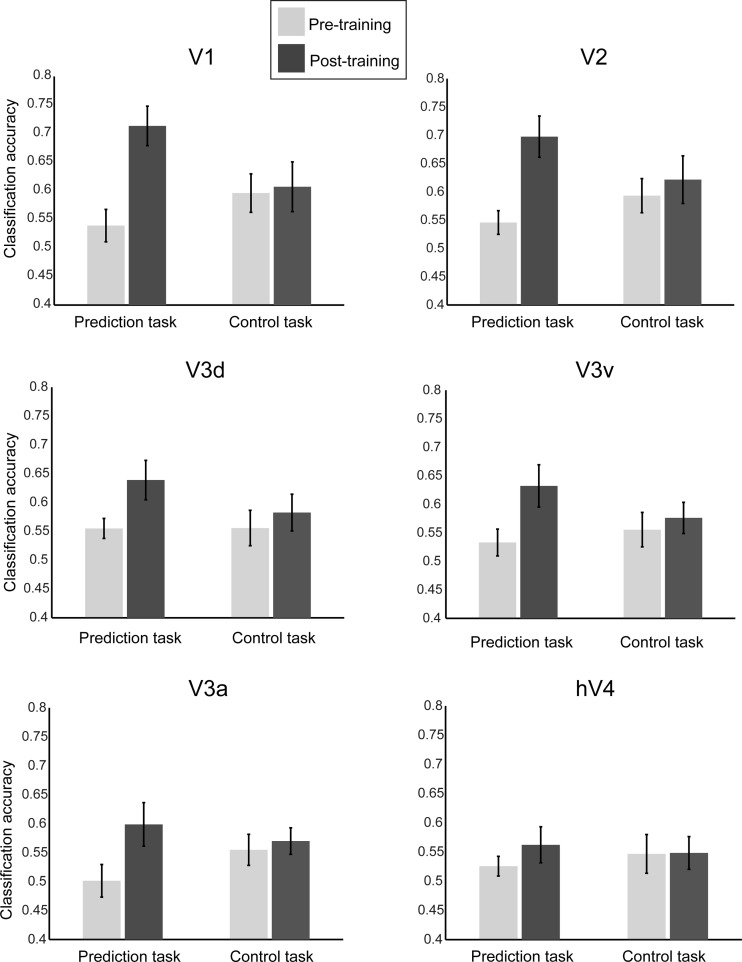
Comparing MVPA for the prediction and control tasks. Mean classification accuracy (across *volumes 8–13* and participants) of the predicted orientation before and after training in early visual areas for the prediction and control tasks. Error bars indicate SE.

To quantify and compare decoding accuracies across visual areas, we selected and averaged fMRI responses from all volumes (*8–13*) that corresponded to the no-stimulation period during which participants predicted the orientation of the upcoming stimulus. Consistent with the behavioral results for the prediction task, decoding of predicted orientations improved significantly after training (Fig. 3). In contrast, for the control task orientation decoding did not change with training, consistent with the participants' behavior in this task.

Comparing decoding accuracies between tasks showed higher decoding accuracies after training for the prediction than the control task. In particular, a 2 (session: pre- vs. posttraining scan) × 2 (task: prediction vs. control) × 6 (ROI: V1, V2, V3d, V3a, V3v, and hV4) repeated-measures ANOVA showed a significant interaction between task and session (*F*_1,11_ = 9.77, *P* = 0.010). Enhanced decoding accuracy after training for the prediction task was primarily observed in early visual areas V1 and V2. In particular, the interaction between task and session was significant in V1 (*F*_1,11_ = 6.683, *P* = 0.025) and V2 (*F*_1,11_ = 6.55, *P* = 0.027), as decoding accuracy increased after training only for the prediction task (paired *t*-test: *t*_11_ = −5.03, *P* < 0.001) but not for the control task (*P* = 0.783). No significant interactions were observed in V3d and V3v and hV4 and V3a (*F* < 1). Furthermore, there was no three-way interaction (session × task × ROI: *F*_5,75_ = 0.545, *P* = 0.741), as indicated by higher classification accuracy in V1 and V2 compared with higher visual areas for both the prediction and the control task. These weaker effects in higher compared with early (V1, V2) visual areas (effect of ROI: *F*_5,55_ = 4.44, *P* = 0.002) have been previously observed in fMRI studies testing responses in nonstimulated visual cortex (Harrison and Tong 2009; Kok et al. 2012, 2013; Smith and Muckli 2010) and are potentially due to stronger orientation-selective responses in early visual areas (Kamitani and Tong 2005).

Furthermore, additional analysis after removal of more volumes at the beginning (*volumes 8* and *9*) and the end (*volume 13*)—to avoid activity due to stimulation from the sequences or the test grating—showed the same pattern of results. As in the main analysis, a 2 (session: pre- vs. posttraining scan) × 2 (task: prediction vs. control) × 6 (ROI: V1, V2, V3d, V3a, V3v, and hV4) repeated-measures ANOVA showed a significant interaction between task and session (*F*_1,11_ = 7.80, *P* = 0.017). Enhanced decoding accuracy after training for the prediction task was primarily observed in early visual areas V1 and V2. That is, we observed significant effects for session in V1 (*F*_1,11_ = 17.77, *P* = 0.001) and V2 (*F*_1,11_ = 7.92, *P* = 0.017) but not in V3d, V3v, V3a, and hV4. There was a significant interaction between session and task in both V1 (*F*_1,11_ = 17.3, *P* = 0.002) and V2 (*F*_1,11_ = 11.39, *P* = 0.006), consistent with enhanced classification accuracy after training only for the prediction task.

These results provide evidence for visual cortex representations that are specific to the learned sequence structure—rather than random sequences as presented in the control task—and reflect participants' predictions. Increased decoding accuracies after training for the prediction task could not be simply explained by *1*) general familiarity with the stimuli or the task after training, as these remained the same across tasks, or *2*) differences in the task design. In particular, as both tasks require a coarse (left vs. right) rather than fine orientation matching of the predicted or cued orientation to the test stimulus, participants may hold in memory and potentially mentally imagine a label or visual image of the predicted or cued stimulus. The cue in the control task may encourage participants to keep a label in memory that can be visualized for comparison to the visual test stimulus, while in the prediction task there is no cue and the participants may verbalize or visualize their prediction. However, this difference in task design did not result in significant differences in average fMRI responses (main effect for task: *F*_1,11_ = 0.01, *P* = 0.905; interaction between session and task: *F*_1,11_ = 0.41, *P* = 0.537) during the no-stimulation period, suggesting similar effect of working memory or imagery processes in early visual cortex across tasks. In contrast, the key difference between tasks is in the content (predicted stimulus following a structured sequence vs. cued physical stimulus following a random sequence) of the representation accessed by the participants. Thus higher decoding accuracies after training in the prediction task compared with the control task—despite similar behavioral performance after training in both tasks (paired *t*-test: *t*_11_ = −1.92, *P* = 0.082)—suggest that our results indicate predictive representations specific to the trained structured sequences rather than differences in working memory or visual imagery processes across tasks.

**Fig. 4.**
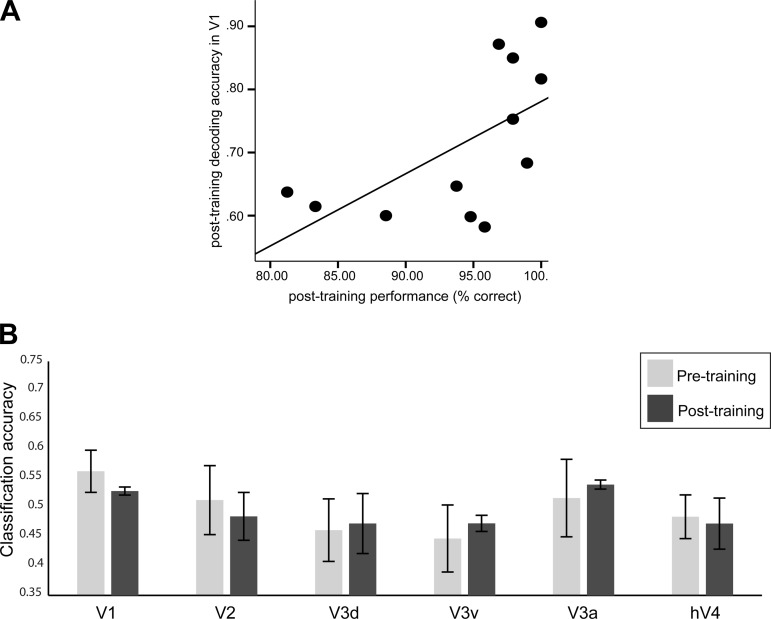
Linking classification accuracy and behavioral performance. *A*: correlation between decoding accuracy for predicted orientations in V1 and performance (% of correct predictions) in the prediction task. No significant correlations (*P* > 0.05) were observed for other visual areas. *B*: decoding accuracy across visual areas for the weak learners (*n* = 4) in the prediction task before and after training.

Finally, significant correlation of the participants' performance with decoding accuracy in V1 after training (*r* = .609, *P* = 0.036) suggests that selective representations for predicted orientation relate to the observers' enhanced ability to predict upcoming stimuli after training on temporal sequences (Fig. 4*A*). Interestingly, decoding accuracy did not improve significantly after training in any of the visual areas (*F* < 1) for participants (*n* = 4) who did not show improved performance during training (57% mean performance at the last training session). A 2 (session: pre- vs. posttraining scan) × 6 (ROI: V1, V2, V3d, V3a, V3v, and hV4) repeated-measures ANOVA showed no significant main effects for session (*F*_1,3_ = 0.028, *P* = 0.878) or ROI or significant interaction (*F*_1,3_ = 0.073, *P* = 0.804) (Fig. 4*B*). Taken together these results suggest a strong link between the observers' ability to predict the identity of upcoming stimuli after training on temporal sequences and orientation representations in early visual areas.

### Comparing Physical vs. Predicted Orientation Representations

**Fig. 5.**
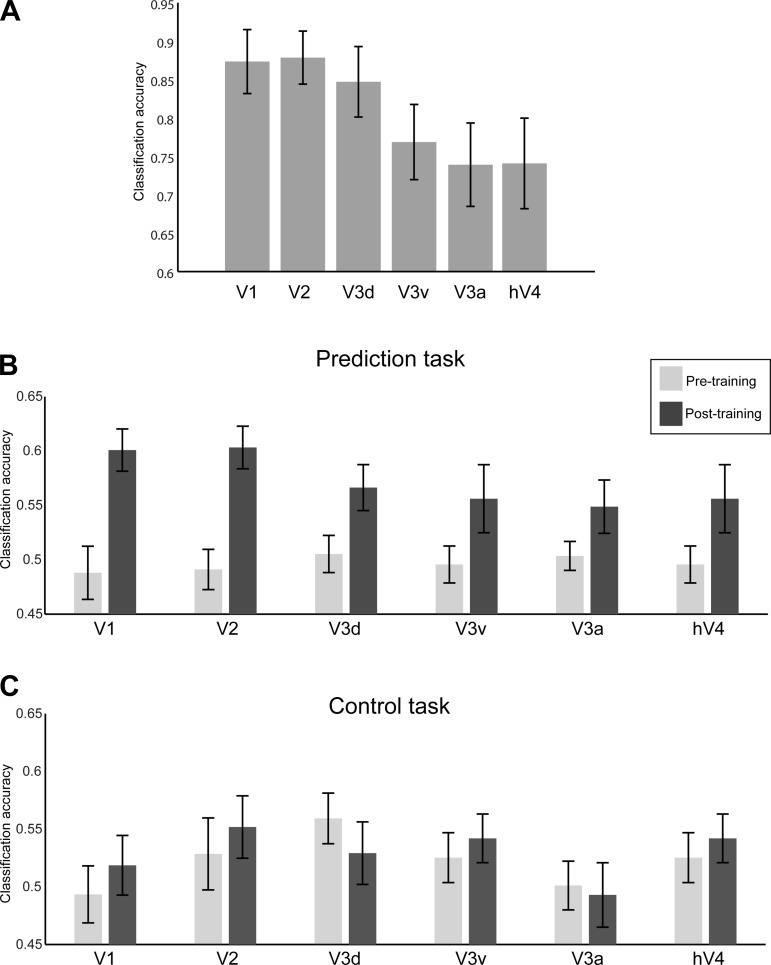
Classifier generalization performance. *A*: classification accuracy for decoding the 2 grating orientations (rightward and leftward) in an independent block design experiment. *B* and *C*: decoding (mean classification accuracy) of predicted (prediction task, *B*) and cued (control task, *C*) orientations before and after training using a classifier trained on physical stimulus orientations.

Finally, we asked whether neural populations—as recorded at a large scale by fMRI—that encode physical stimulus orientation may also represent predicted orientation. To this end, we tested whether activity patterns for predicted orientations resemble stimulus-driven activity elicited during viewing of oriented gratings. We collected an independent set of fMRI data while the participants viewed leftward- vs. rightward-oriented gratings in separate blocks (45° or 135°). Consistent with previous studies (Harrison and Tong 2009; Haynes and Rees 2005; Kamitani and Tong 2005), decoding of orientation from stimulus-driven activity was successful across visual areas (Fig. 5*A*). To evaluate the correspondence between neural representations for physical and predicted orientations, we trained an SVM on fMRI signals related to physical orientations and tested the accuracy of the classifier in decoding predicted orientations from fMRI data collected when observers performed the prediction task. Despite stimulus and task differences between these experiments, we observed generalization of classifier performance in V1 and V2 after training but not in higher areas where orientation selectivity is known to be weaker. Importantly, we observed improved classification accuracy for predicted orientations after training, suggesting that learning temporal sequences modulates neural populations in early visual cortex involved in the selective processing of orientation (Fig. 5*B*). In particular, a 2 (session: pre- vs. posttraining) × 6 (ROI: V1, V2, V3d, V3a, V3v, and hV4) repeated-measures ANOVA showed higher classification accuracy after training (i.e., main effect for session: *F*_1,11_ = 18.768, *P* = 0.001). Although there was no significant interaction between session and ROI (*F*_1,11_ = 1.05, *P* = 0.400), improved decoding accuracy after compared with before training was more prominent in V1 (paired *t*-test: *t*_11_ = −3.62, *P* = 0.004) and V2 (paired *t*-test: *t*_11_ = −4.16, *P* = 0.002) but only marginally significant for V3d (paired *t*-test: *t*_11_ = −1.93, *P* = 0.080). Similar analysis for the control task (Fig. 5*C*) did not show any significant differences after vs. before training in predicting cued orientations across ROIs [paired *t*-tests: *t*_11_ < 1, not significant (n.s.)]. Comparing generalization of classifier performance between the prediction and control tasks showed a significant interaction between task and session (*F*_1,11_ = 5.17, *P* = 0.044), consistent with our main findings suggesting enhanced predictive representations that are specific to training with structured than random sequences of items.

### Control Analyses

We conducted the following additional analyses to control for possible alternative explanations of the results.

First, to control for the possibility that our results are due to random correlations in the data, we conducted the same decoding analysis using randomly permuted fMRI patterns (i.e., we randomized the correspondence between fMRI data and labels and performed MVPA for 10,000 iterations). This analysis resulted in classification accuracies that did not differ from chance for both pretraining (mean = 50%, SD = 0.05) and posttraining (mean = 49.8%, SD = 0.054) data (*t* < 1, n.s.) in V1. In particular, decoding accuracy for the prediction task was higher than the 97.5th percentile of the random distribution for 10 of 12 participants after training but only for 2 participants before training. Similar results were observed across ROIs. This analysis suggests that our decoding results could not be simply accounted for by random variations in the data.

**Fig. 6.**
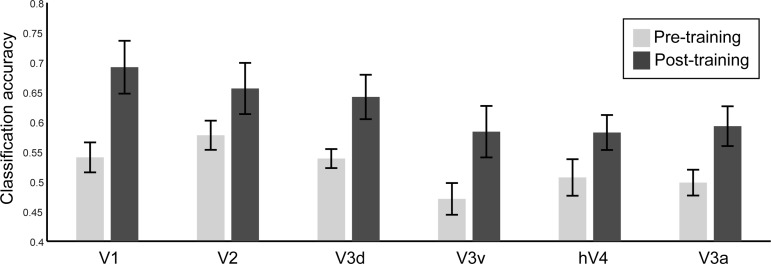
Decoding the expected orientation as determined by the preceding sequence. Mean classification accuracy (across *volumes 8–13* and participants) of decoding the expected orientation as determined by the presented sequence before and after training in early visual areas. Error bars indicate SE.

Second, we focused on fMRI decoding based on the participants' responses in the prediction task, as our goal was to understand whether the visual cortex contains information that relates to behavioral predictions. However, this analysis may be confounded by differences in the number of correct trials between sessions, that is, a larger number of correct trials after compared with before training. To control for this, we first conducted the same analysis using fMRI signals only for correct trials for both scan sessions. A 2 (session: pre- vs. posttraining scan) × 2 (task: prediction vs. control) × 6 (ROI: V1, V2, V3d, V3a, V3v, and hV4) repeated-measures ANOVA showed similar improvement of classification accuracies after training. That is, we observed significantly higher classification accuracy after than before training in the main task as indicated by significant session × task interactions for V1 (*F*_1,11_ = 20.59, *P* = 0.001), V2 (*F*_1,11_ = 15.43, *P* = 0.002), V3a (*F*_1,11_ = 8.83, *P* = 0.013), and V3d (*F*_1,11_ = 5.26, *P* = 0.043) but not for V3v (*F*_1,11_ = 3.056, *P* = 0.108) and hV4 (*F*_1,11_ = 0.356, *P* = 0.563). Second, we performed an additional analysis (Fig. 6) that decoded the expected orientation as determined by the presented sequence rather than the participants' prediction. We found a similar pattern of results, with significantly higher classification accuracies after than before training across ROIs (session × task: *F*_1,11_ = 10.76, *P* = 0.007). Interestingly, this was not the case for weaker learners: prediction accuracies did not change across sessions, consistent with the results presented in Fig. 4*B*. These control analyses suggest that our results could not be simply explained by differences in task difficulty between scanning sessions.

Third, is it possible that the difference in the decoding accuracy for the prediction task before and after training was due to different strategies adopted by the observers? That is, before training the participants found the task too difficult and responded randomly when the test stimulus appeared instead of predicting the upcoming stimulus. However, low prediction accuracies before training for the expected orientation as determined by the presented sequence (Fig. 6) suggest that fMRI patterns in visual cortex contain information about structured sequences after but not before training. As this analysis does not rely on the participants' responses, low accuracies before training could not be due to the participants' response strategy (e.g., guessing).

Fourth, our results could not be simply explained by differences in participants' attention between scan sessions, as analysis of univariate fMRI signals for leftward- and rightward-oriented gratings did not differ before and after training. Furthermore, this analysis justifies our choice of MVPA classification methods for the analysis of predictive representations that have been shown to be more sensitive than univariate approaches in extracting selective signals from brain patterns. Finally, analysis of eye movement recordings did not show any differences between scanning sessions and between leftward vs. rightward predicted orientations across sessions, suggesting that it is unlikely that eye movements could explain our results. In particular, a 2 (session: pre- vs. posttraining) × 2 (orientation: left vs. right) repeated-measures ANOVA revealed that there were no significant effects of session or orientation for the mean eye position (session: *F*_1,3_ = 0.086, *P* = 0.789; orientation: *F*_1,3_ = 0.29, *P* = 0.625), mean saccade amplitude (*F*_1,3_ = 2.2, *P* = 0.230; *F*_1,3_ = 0.48, *P* = 0.536), or number of saccades per trial per condition (*F*_1,3_ = 0.57, *P* = 0.504; *F*_1,3_ = 0.42, *P* = 0.564). Also, there was no significant interaction between session and orientation for the mean eye position (*F*_1,3_ = 1.89, *P* = 0.263), the number of saccades (*F*_1,3_ = 0.55, *P* = 0.512), or the saccade amplitude (*F*_1,3_ = 0.44, *P* = 0.55).

## DISCUSSION

Our results provide evidence that learning of temporal regularities supports our ability to predict future events by reactivating selective sensory representations in primary visual cortex. Interestingly, these predictive representations appear to be driven by the same large-scale neural populations that encode physical stimulus properties (i.e., orientation) and to be specific to the learned sequence structure. Furthermore, these representations reflect our ability to predict future events as indicated by a significant correlation between fMRI decoding and behavioral improvement in the prediction task after training.

Consistent with our previous behavioral work (Baker et al. 2014), we demonstrate that exposure to temporal regularities in a scene allows us to accumulate information about its structure and predict future events. Although we used deterministic sequences, we ensured that observers learned the global sequence structure (i.e., temporal order statistics across items rather than each item position in the sequence) by matching the frequency of occurrence of each item (i.e., grating orientation) in the sequence. Previous studies have suggested that learning of regularities may occur implicitly in a range of tasks: visuomotor sequence learning (Nissen and Bullemer 1987), artificial grammar learning (Reber 1967), probabilistic category learning (Knowlton et al. 1994), and contextual cue learning (Chun and Jiang 1998). In our study, participants were exposed to the sequences without feedback but were asked to make an explicit judgment about the identity of the upcoming test stimulus (leftward- vs. rightward-oriented grating), making them aware of the dependencies between the stimuli presented in the sequence. However, debriefing the participants showed that it was unlikely that the participants explicitly memorized the sequences, suggesting that they made predictions based on implicit knowledge of temporal structure.

Our fMRI findings advance our understanding of the brain mechanisms that support our ability to translate previous knowledge to future predictions in four main respects. First, previous imaging and physiology studies suggest that extensive training on visual detection or discrimination tasks may modulate processing in primary visual cortex (Bao et al. 2010; Furmanski et al. 2004; Jehee et al. 2012; Schoups et al. 2001). Our findings extend beyond this work, showing that mere exposure to the statistics of the environment alters selectively orientation representations in primary visual cortex to reflect the observers' prediction. Furthermore, recent animal physiology studies provide evidence for reactivation of neural responses in visual cortex when neurons are activated in a temporal sequence (Eagleman and Dragoi 2012; Gavornik and Bear 2014). Our study provides novel evidence that such experience-dependent neural reactivation correlates and may facilitate the ability of human observers to make predictions of upcoming sensory events. Although the nature of the signals that support orientation decoding has been recently debated (Freeman et al. 2011), here we demonstrate that the same large-scale neural populations that encode physical orientations in primary visual cortex encode also the predicted orientations. Thus our work provides novel evidence that previous knowledge alters processing in primary visual cortex that mediates our ability to make sensory predictions.

Second, learning of spatial and temporal regularities has been suggested to engage the striatum and medial temporal lobe regions (Gheysen et al. 2011; Hsieh et al. 2014; Rauch et al. 1997; Rose et al. 2011; Schapiro et al. 2012, 2014; Schendan et al. 2003a). For example, studies (Foerde et al. 2006; Poldrack et al. 1999, 2001) using the weather prediction paradigm have implicated these regions in implicit learning of probabilistic associations. Previous work has implicated mainly striatal regions (e.g., caudate and putamen) in implicit learning (Hazeltine et al. 1997; Rauch et al. 1995) and the medial temporal lobe in both implicit and explicit learning (Schendan et al. 2003a, 2003b). Our results suggest interactions between these memory circuits and visual cortex; that is, learning of temporal structures that is known to engage this circuit may shape representations in primary visual cortex that relate to our ability to make sensory predictions. This is consistent with recent work implicating the hippocampus in memory of temporal order (for review see Eichenbaum 2013; Howard and Eichenbaum 2013) and prospective memory of simulated future events (Ingvar 1985; Szpunar et al. 2013). Furthermore, related work has suggested that associative learning engaging medial temporal lobe regions modulates processing in inferotemporal (Meyer and Olson 2011; Miyashita 1993) and area MT (Schlack and Albright 2007) and early visual (Bosch et al. 2014) cortex. While this previous work has focused on paired associations, we propose that learning of higher-order regularities in the context of temporal sequences may employ similar brain circuits to translate knowledge about temporal structure in medial temporal lobe to predictions in sensory areas.

Third, there is accumulating evidence for the role of primary visual cortex in predictive coding. In particular, recent fMRI (Alink et al. 2010; Harrison et al. 2007; Kok et al. 2012, 2014; Murray et al. 2002; Schoups et al. 2001; Smith and Muckli 2010; Summerfield and Egner 2009) and neurophysiology (Guo et al. 2007; Kim et al. 2012; Meyer and Olson 2011; Perrett et al. 2009) studies have shown that responses in visual cortex are modulated by spatio-temporal context. These findings have been observed in the context of tasks involving stimulus anticipation based on paired associations or short-term history (i.e., probability of occurrence for a preceding stimulus). Furthermore, higher responses are observed for unexpected than expected stimuli, consistent with increased prediction error when sensory signals and top-down expectations do not correspond (Bastos et al. 2012; Friston 2005; Summerfield and Egner 2009). Our study extends beyond these findings in several respects. First, our paradigm allows us to test how longer-term knowledge acquired through several training sessions rather than short-term stimulus history affects prediction in primary visual cortex. Second, our study is the first to test the role of sequence learning on predictions related to visual recognition. Previous work on learning temporal sequences has focused on implicit measures of sequence learning, such as familiarity judgments or reaction times (Nissen and Bullemer 1987; for review see Schwarb and Schumacher 2012). Although such paradigms implicate that implicit learning of temporal sequences facilitates the anticipation of upcoming events, they do not test whether this knowledge can be used to explicitly predict the identity of upcoming stimuli. In contrast, our design allows us to test for neural representations related to explicit predictions about the identity of an upcoming stimulus (i.e., grating orientation) rather than anticipation as revealed typically by implicit measures (e.g., reaction times, familiarity) of visual recognition. Third, using MVPA classification methods allows us to test how previous knowledge affects selective processing of sensory features (i.e., orientation) related to the observers' response (i.e., per-trial prediction) rather than simply changes in the overall fMRI magnitude related to expectation. Decoding predicted orientation during a no-stimulation period before the test stimulus appears allows us to investigate the processes involved in predicting upcoming sensory events, in contrast to previous work investigating predictive coding based on the error generated when unexpected stimuli are presented.

Finally, recent imaging work has highlighted the role of primary visual cortex in cognitive functions such as working memory and visual imagery (Albers et al. 2013; Harrison and Tong 2009; Serences et al. 2009). The prediction task used in our study involves these processes, as it entails that participants hold in memory and/or imagine the predicted stimulus in order to match it to the test stimulus. However, comparing the prediction task with a control task using random sequences and a similar design involving the same processes (i.e., holding in memory and/or imagining a grating orientation) demonstrates predictive representations in primary visual cortex that are specific to the knowledge of structured sequences. In particular, comparing average fMRI responses for the no-stimulation period between tasks did not show any significant differences, suggesting that higher orientation decoding accuracy in the prediction than the control task cannot be simply due to differences in the task design. In contrast, the critical difference between tasks is in the content of the representation accessed by the participants (predicted orientation following a structured sequence vs. cued physical orientation following a random sequence), which we decode using MVPA classification of fMRI data. This result is further supported by significant correlation of the participants' performance in the prediction task with decoding accuracy in V1 after training. Taken together these results suggest predictive representations in early visual cortex following learning of structured sequences that cannot be simply explained by differences in working memory or visual imagery processes across tasks.

In sum, our findings provide evidence that knowledge of temporal regularities alters processing in primary visual cortex to support our ability for sensory predictions. The high-resolution imaging adopted in our study afforded us the signal quality necessary to reveal activity patterns related to predictive representations, but it restricted brain coverage to visual cortex. Given the complex nature of the BOLD signal, it is possible that the fMRI selectivity that we observed for predicted orientations is enhanced by feedback from other cortical circuits. Possible candidates include *1*) medial temporal lobe and subcortical areas that are known to be involved in associative learning and temporal memory and *2*) prefrontal circuits that support rule-based behaviors and prediction of future events (Bar 2009; Leaver et al. 2009; Pasupathy and Miller 2005). It is also important to note that—despite the enhanced sensitivity of our methodology—decoding reveals neural preferences at the scale of large neural populations rather than tuning of individual neurons. Therefore, understanding the cortical circuits that support our ability to translate previous knowledge to sensory predictions requires further whole brain connectivity studies combining advanced imaging and neurophysiological techniques.

## GRANTS

This work was supported by a Wellcome Trust Senior Research Fellowship to A. E. Welchman (095183/Z/10/Z) and grants to Z. Kourtzi from the Biotechnology and Biological Sciences Research Council (H012508), a Leverhulme Trust Research Fellowship (RF-2011-378), and the People Programme (Marie Curie Actions) of the European Union's Seventh Framework Programme FP7/2007–2013/under REA grant agreement no. PITN-GA-2011-290011.

## DISCLOSURES

No conflicts of interest, financial or otherwise, are declared by the author(s).

## AUTHOR CONTRIBUTIONS

Author contributions: C.D.B.L., A.E.W., and Z.K. conception and design of research; C.D.B.L. performed experiments; C.D.B.L. and A.M. analyzed data; C.D.B.L., A.M., A.E.W., and Z.K. interpreted results of experiments; C.D.B.L., A.M., and Z.K. prepared figures; C.D.B.L., A.M., and Z.K. drafted manuscript; C.D.B.L., A.M., A.E.W., and Z.K. edited and revised manuscript; C.D.B.L., A.M., A.E.W., and Z.K. approved final version of manuscript.
